# Impact of changing from staining to culture techniques on detection rates of *Campylobacter* spp. in routine stool samples in Chile

**DOI:** 10.1186/s12879-016-1546-7

**Published:** 2016-05-13

**Authors:** Lorena Porte, Carmen Varela, Thomas Haecker, Sara Morales, Thomas Weitzel

**Affiliations:** Laboratorio Clínico, Clínica Alemana de Santiago, Facultad de Medicina Clínica Alemana, Universidad del Desarrollo, Av. Vitacura 5951, Santiago, Chile

**Keywords:** *Campylobacter*, Diagnosis, Culture media, Staining methods

## Abstract

**Background:**

*Campylobacter* is a leading cause of bacterial gastroenteritis, but sensitive diagnostic methods such as culture are expensive and often not available in resource limited settings. Therefore, direct staining techniques have been developed as a practical and economical alternative. We analyzed the impact of replacing *Campylobacter* staining with culture for routine stool examinations in a private hospital in Chile.

**Methods:**

From January to April 2014, a total of 750 consecutive stool samples were examined in parallel by Hucker stain and *Campylobacter* culture. Isolation rates of *Campylobacter* were determined and the performance of staining was evaluated against culture as the gold standard. Besides, isolation rates of *Campylobacter* and other enteric pathogens were compared to those of past years.

**Results:**

*Campylobacter* was isolated by culture in 46 of 750 (6.1 %) stool samples. Direct staining only identified three samples as *Campylobacter* positive and reached sensitivity and specificity values of 6.5 and 100 %, respectively. In comparison to staining-based detection rates of previous years, we observed a significant increase of *Campylobacter* cases in our patients.

**Conclusion:**

Direct staining technique for *Campylobacter* had a very low sensitivity compared to culture. Staining methods might lead to a high rate of false negative results and an underestimation of the importance of campylobacteriosis. With the inclusion of *Campylobacter* culture, this pathogen became a leading cause of intestinal infection in our patient population.

## Background

The genus *Campylobacter* comprises fastidious S-shaped or spiral gram-negative bacteria with a length of 0.5 to 5 μm, which are microaerophilic, non-spore-forming, and mobile by the presence of a polar flagellum. *Campylobacter* is among the most important causes of foodborne infections causing human gastroenteritis worldwide [1]. Infections with this pathogen might be followed by severe sequelae such as reactive arthritis, Guillain-Barré syndrome, and irritable bowel syndrome [2]. *Campylobacter* species are ubiquitous in the environment and the intestinal tracts of a variety of free-living birds, wild and domestic food animals, and pets [3, 4]. As an emerging zoonotic agent, it is of growing public health importance and also affects non-clinical areas such as food-production and animal handling [1].

There has been a rise in the incidence of campylobacteriosis in the last ten years in most developed regions such as North America, Europe, and Australia [5]. In South America, the role of *Campylobacter* as an enteric pathogen is less clear, since epidemiological data are scant and inconclusive [1, 5, 6]. In Chile, *Campylobacter* is a notifiable enteric pathogen. Recently, the National Reference Laboratory (Instituto de Salud Pública, Santiago, Chile) reported an average of 91 annual *Campylobacter* cases for the whole of Chile during 2005 to 2012, implicating incidence rates of 0.1 to 0.6/100.000 [7]. In comparison, the current rate in the USA is 13.5/100.000 [8]. A recent study from southern Chile detected *C. jejuni* in 10 and 10.7 % of diarrheic samples by culture and PCR, respectively [9]. Older reports from the 1980s and 1990s, which mainly focused on pediatric populations, showed incidence rates of 10 and 16 % in symptomatic children [10, 11].

Culture methods are the gold standard for the diagnosis of intestinal *Campylobacter* infections. However, these methods require special selective media incubated at 37° and/or 42 ° C under microaerophilic conditions and are therefore inconvenient and expensive [12]. As a consequence, they are not included within the routine stool workup of many non-industrialized countries including Chile [7]. As a more economical alternative, direct stool staining methods have been developed and currently, these techniques are recommended in Chile for the routine detection of *Campylobacter* in stool samples of patients with acute gastroenteritis [13]. However, these methods are insufficiently evaluated and have drawbacks such as operator dependency. Due to sensitivity problems [14], they might underestimate the true incidence of this organism.

The aim of this study was to compare *Campylobacter* detection rates by microscopic examination to those based on culture in routine stool samples and to analyze the possible impact of performing *Campylobacter* culture as a routine method on the epidemiological data of campylobacteriosis in our clinical setting.

## Methods

The study was conducted between January and April 2014 in the Clinical Laboratory of Clínica Alemana in Santiago, Chile. It utilized consecutive stool samples that were tested as part of routine care, which included culture methods for *Salmonella*, *Shigella*, *Yersinia*, and *Vibrio*, as well as direct stain for *Campylobacter.* Samples consisted of fresh stool, transported at ambient temperature and processed within 2 h after collection. For culture and enrichment, commercial selective media such as Mac Conkey, *Salmonella-Shigella* agar, CIN, TCBS, selenite broth, alkaline peptone water (all bioMérieux, l’Etoile, France) were used following standard recommendations [2, 15, 16]. *Campylobacter* staining was performed as recommended in Chile [13] and previously described [14, 17]. In short, smears of undiluted stool were prepared using a swab (155C, Copan, Brescia, Italy) and air dried. Then, slides were flooded for 1–2 min with a monthly prepared solution of equal parts of 1 % sodium bicarbonate and commercial crystal violet solution (Color Gram 2 R1, bioMérieux). Slides were examined during routine workflow by a trained technician (50 high power oil immersion fields) for the presence of S-shaped or spiral rods. For quality control, all positive or doubtful slides were sent to the National Reference Laboratory. Each sample was inoculated with a swab on *Campylobacter* culture medium (Campylosel agar, bioMérieux), streaked into 4 quadrants with a sterile loop and incubated at 42 °C under microaerobic conditions (Anaerocult® C, Merck, Darmstadt, Germany). Quality control for each batch of culture medium was performed using *C. jejuni* ATCC 33291 (growth at 48 h) and *E. coli* ATCC 25922 (growth inhibition) as recommended [18]. *Campylobacter* plates were read after 48 h and suspicious colonies were further identified by matrix-assisted laser desorption/ionization time-of-flight (MALDI-TOF) technology using VITEK MS (bioMérieux). Following surveillance regulations, all enteropathogens were sent to the National Reference Laboratory for confirmation and susceptibility testing. *Campylobacter* strains were studied against ciprofloxacin, erythromycin and tetracycline by E-Test (bioMérieux) on blood Mueller-Hinton agar incubated for 24 h at 42 °C under microaerobic conditions. Results were interpreted according to CLSI guidelines [19].

The performance of *Campylobacter* staining technique was evaluated using culture as gold standard. To estimate the impact of culture methods on the detection of *Campylobacter*, isolation rates of all enteric bacterial pathogens within the study period were compared to those of the same time span of past years in our laboratory. These data were obtained from the laboratory databases. To compare isolation rates, 95 % confidence intervals were calculated and the Z test for comparing two independent proportions was applied. *P* values <0.05 were considered significant. For statistical analysis, Clinical Calculator 1 (http://vassarstats.net) was used.

## Results

A total of 750 stool samples were examined. In 96 of them (12.8 %), bacterial pathogens were detected (Table 1). All enteric pathogens were identified to the species level with high identification scores (99.9 %) by Vitek MS.

**Table 1:** Number and rate of bacterial pathogens identified in 750 routine stool samples

Pathogen	n	Isolation rate (%)	CI 95 %
*Salmonella enterica*	49	6.5	4.9–8.6
Enteritidis	33		
Montevideo	7		
Heidelberg	2		
Saintpaul	2		
Sandiego	1		
Othmarschen	1		
Infantis	1		
Derby	1		
Not typed	1		
*Campylobacter* (by culture)	46	6.1	4.6–8.1
*C. jejuni*	41		
*C. coli*	5		
*Campylobacter* spp. (by staining)	3	0.4	0.1–1.3
*Yersinia enterocolitica*	1	0.1	0.01–0.9
*Vibrio parahaemolyticus*	1	0.1	0.01–0.9
Total	96^a^	12.8	10.5–15.5

^a^Including one sample with double infection, *C. jejuni* and *Y. enterocolitica*

*Campylobacter* culture was positive in 46 (6.1 %) samples. The median age of the *Campylobacter* positive patients was 12 years (range: 1 to 89). The majority (41/46; 89.1 %) of strains was *C. jejuni*, the other 10.9 % were *C. coli*. Staining technique identified three samples as *Campylobacter* positive, all of which contained *C. jejuni*. Thus, the *Campylobacter* detection rate using Hucker stain was 0.4 % (Table 1) with a sensitivity of 6.5 % (CI95 %: 1.7–18.9) and a specificity of 100 % (CI95 %: 99.3–100). Positive and negative predictive values for staining were 100 % (CI95 % 31.0–100) and 94.2 % (CI95 % 92.3–95.8), respectively.

**Fig. 1:**
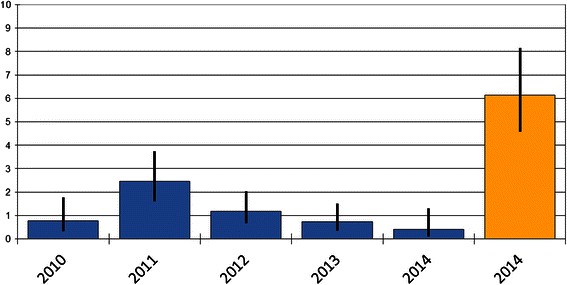
Detection rates of *Campylobacter* in routine stool samples. Graph showing percentage of *Campylobacter* positive samples within January to April of the respective year. *Blue* columns represent rates based on *Campylobacter* direct staining, orange column on *Campylobacter* culture. *Black* bars indicate the 95 % confidence intervals. *P* values by Z test for comparing two independent proportions

**Fig. 2:**
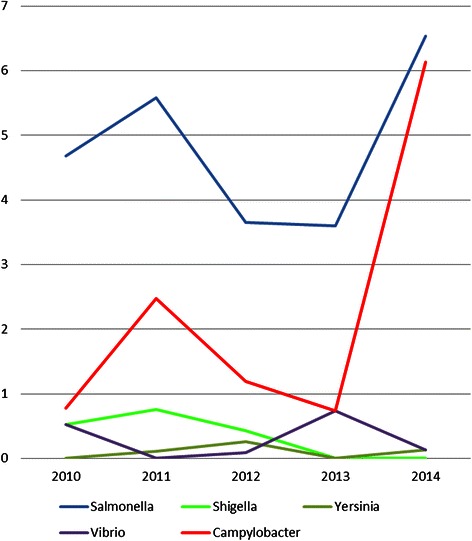
Detection rates of enteric bacterial pathogens in routine stool samples. Graph showing percentage of enteropathogenic bacteria detected in stool samples within January to April of the respective year. All numbers are based on culture techniques except *Campylobacter* rates of the years 2010 to 2013, which are based on staining method

The culture-based *Campylobacter* rate within our study period (January to April 2014) was significantly higher than the staining-based rates of previous years (Fig. 1). A comparison of detection rates of all bacterial enteropathogens identified in stool samples in our laboratory in the months of January to April of 2010 to 2014 demonstrated a clear change of the epidemiological relevance of *Campylobacter* after the introduction of culture methods in 2014 (Fig. 2). In the years 2010 to 2013, *Salmonella* was clearly the most frequently isolated enteropathogen, whereas the other bacterial causes were only rarely detected. In 2014, with the introduction of *Campylobacter* culture, *Campylobacter* became, together with *Salmonella,* the leading cause of bacterial enteritis in our patient collective.

National Reference Laboratory studied fourteen of the Campylobacter strains for antimicrobial susceptibility. Of those, 13 (92.8 %) were susceptible to erythromycin, whereas tetracycline- and ciprofloxacin-resistance was detected in three (21.4 %) and five (35.7 %) of the isolates, respectively.

## Discussion

*Campylobacter* is a leading cause of gastrointestinal infections in industrialized and non-industrialized countries [1]. Its clinical and epidemiological importance is underlined by the ubiquitous nature of the pathogen, the possibility of severe post-infectious sequelae, and the increase of its burden during the last decade. Unfortunately, underreporting of campylobacteriosis is common and in some regions, the epidemiology of this infection is incompletely understood [5].

In most South American countries, the epidemiological role of *Campylobacter* as an enteric pathogen is unknown, since systematic studies are scant and national surveillance programs are mostly absent [1, 5]. The main reason for this lack of data is that *Campylobacter* culture is expensive or not available and therefore, rarely included in the routine stool workup in microbiological laboratories [14]. In Chile, enteric *Campylobacter* infections were included in the national mandatory decree of active laboratory surveillance of enteropathogens in 1993 [7]. Since then, due to financial limitations, most Chilean laboratories adopted staining methods such as Hucker stain as the routine diagnostic tool for *Campylobacter* as suggested by local recommendations [13, 14]. The presented study highlights a potential problem of this approach since direct stool microscopy proved to have a very low and insufficient sensitivity compared to culture. In a previous study using the same technique in Chile, the sensitivity was higher, but the difference was not statistically significant (37.5 %, CI95 % 16.3–64.1) [14]. Other studies using different stains such as Gram, reported much higher sensitivity values from 44 to 94 % [20–24]. Reasons for the lower sensitivity in our study might be the use of a different staining technique, diagnostic settings (study versus routine conditions), study populations (children versus adults), and technical problems with *Campylobacter* culture, especially in older reports or in studies from developing countries. A main obstacle is that direct staining methods are highly operator dependent because they require significant expertise [2]. Therefore, objective evaluations of the performance are difficult and some experts discourage the routine use of these methods [15]. Another obstacle for a standardization of these techniques is the lack of protocols for quality control. External quality control programs offered from international institutions such as the College of American Pathologists (CAP) do not include direct *Campylobacter* staining. To overcome this limitation, microscopists should regularly be re-trained and tested. In accordance with other reports, the specificity of *Campylobacter* staining in our study was high [14, 20–24]. Positive results therefore, provide rapid and clinically relevant information. Still, direct staining can only serve as a supplemental test and should not replace *Campylobacter* culture.

Besides the costs, a drawback of *Campylobacter* culture is the prolonged incubation period. Still, the optimum time is controversial. While in North America, 72 h of incubation are recommended [2], British and German guidelines suggest shorter incubation periods of 40–48 h [25, 26]. As a recent survey of more than 400 laboratories in the USA revealed, most participants also used 48 h for routine incubation for *Campylobacter* culture [27]. In our study we incubated cultures for 48 h as currently recommended by the Chilean consensus statement [13].

Culture-independent methods for *Campylobacter* diagnosis are commercially available in many countries in the industrialized world. Such tests include the detection of *Campylobacter* specific antigen and nucleic acids in stool samples. Until now, these tests are insufficiently evaluated to replace the traditional culture methods and do not serve for susceptibility testing and public health surveillance purposes [2]. The WHO encourages further research to validate the usefulness of these tests in low- and middle-income countries [1]. A recent study with samples from Tanzania, Bangladesh, and Peru showed higher *Campylobacter* prevalence rates than with culture, but no clear association with diarrheal disease [28]. In Chile, PCR detected higher prevalence of campylobacteriosis than culture, but most of the additional cases were caused by emerging *Campylobacter* species such as *C. concisus* [9]. To our opinion, the introduction of such new culture-independent techniques in countries with uncertain epidemiology such as Chile seems problematic at present.

With the inclusion of culture methods in routine stool examination during the study period, the *Campylobacter* detection rate increased by more than 10-fold compared to staining and was also significantly higher than in the same time period of the previous years (Fig. 1). Since a comparison of the staining-based rates did not show any evidence of an epidemiological change, we assume that the emergence of *Campylobacter* was truly related to the implementation of the new diagnostic procedure. A comparison of the detection rates of all bacterial enteropathogens during the years of 2010 to 2014 revealed that after the inclusion of culture, *Campylobacter* became a leading enteric pathogen with rates similar to those of *Salmonella*. This was a surprising finding, since in the official surveillance reports of recent years, *Salmonella* largely exceeds *Campylobacter*. In 2011 and 2012, for example, the respective numbers of cases were 3627 and 3076 for *Salmonella*, but only 170 and 136 for *Campylobacter* [7]. We believe that these extremely low rates of *Campylobacter* isolates might rather reflect on the insufficient diagnostic capacities to detect this pathogen than on the true epidemiological situation. This misjudgment and lack of reliable data leads to an underestimation of the importance of *Campylobacter* and further ignorance regarding its diagnosis - an epidemiological vicious circle. To our opinion, the implementation of *Campylobacter* culture as the routine diagnostic method in Chile is recommendable, although an exact analysis of the cost-effectiveness of this method is pending.

Matrix-assisted laser desorption/ionization time-of-flight (MALDI TOF) proved to be a rapid, convenient, and reliable diagnostic tool for *Campylobacter* species identification. If available, this technology might help to better understand the distribution of species other than *C. jejuni*. In our study, *C. coli* was less frequently isolated (5/46; 10.9 %) than previously reported in diarrhea cases in southern Chile (27.7 %) [6].

Our data confirmed the high percentage of ciprofloxacin resistance of *Campylobacter jejuni* in Chile, which has previously been reported [29]. Surprisingly, one of the strains was also resistant to erythromycin, which had not been reported before in strains of human origin in Chile. These findings support the need for routine *Campylobacter* culture and for further surveillance of resistance.

The age distribution of our patients differed from older data in Chile, which reported that the majority of cases occurred in infants and preschool children [14], but was in accordance with a more recent study from southern Chile [9]. We observed a median age of 12 years without predominance of children less than 5 years. This might be explained by the high socioeconomic status of our patients attending a private hospital, since in North America and Europe campylobacteriosis in individuals older than ten years was associated with higher socioeconomic conditions [30].

Limitations of this study were that it covered only a limited period of time and samples derived only from a single clinical center. This might have aggravated the operator dependency of the staining method. As a study based on routine samples, the clinical data were limited. Furthermore, we did not include the study of viral or parasitic etiologies of enteritis.

## Conclusions

Our study showed that with the inclusion of *Campylobacter* culture in the routine bacteriological stool workup, this pathogen became a leading cause of intestinal infection in our patient population. Direct *Campylobacter* staining methods, which have been promoted for resource-limited settings, proved to be of low sensitivity and lead to a significant underestimation of the incidence of campylobacteriosis.

### Ethics statement

The study was approved by the Comité de Ética de la Investigación (N° 2014–73), Centro de Bioética, Universidad del Desarrollo-Clínica Alemana, Santiago, Chile. Informed consent was waived since the study was performed within routine diagnostic procedures and did not involve intervention or interactions with patients.

### Consent to publish

Not applicable.

### Availability of data and materials

All the data supporting our findings is contained within the manuscript.

## References

[1] WHO. The Global view of campylobacteriosis, report of an expert consultation. Utrecht, Netherlands, 2012. http://www.who.int/iris/bitstream/10665/80751/1/9789241564601_eng.pdf. Accessed 20 Aug 2015.

[2] Fitzgerald C, Nachamkin I, Jorgensen JH, Pfaller MA (2015). *Campylobacter* and *Arcobacter*. Manual of clinical microbiology.

[3] Ramonaite S, Kudirkiene E, Tamuleviciene E, Leviniene G, Malakauskas A, Gölz G (2014). Prevalence and genotypes of *Campylobacter jejuni* from urban environmental sources in comparison with clinical isolates from children. J Med Microbiol.

[4] Enberg J (2006). Contributions to the epidemiology of *Campylobacter* infections. Dan Med Bull.

[5] Kaakoush N, Castaño-Rodríguez N, Mitchell H, Man SM (2015). Global epidemiology of *Campylobacter* infection. Clin Microbiol Rev.

[6] Fernández H (2011). *Campylobacter* and campylobacteriosis: a view from South America. Rev Peru Med Exp Salud Publica.

[7] Instituto de Salud Pública de Chile. Vigilancia de laboratorio de *Campylobacter* sp. Chile, 2005 – 2013. In: Boletín Instituto de Salud Pública, Vol. 4, No. 1, Enero 2014. http://www.ispch.cl/sites/default/files/Boletín%20Campylobacter.pdf. Accessed 20 Aug 2015.

[8] Crim SM, Griffin PM, Tauxe R, Marder EP, Gilliss D, Cronquist AB (2015). Centers for Disease Control and Prevention (CDC). Preliminary incidence and trends of infection with pathogens transmitted commonly through food - Foodborne Diseases Active Surveillance Network, 10 U.S. sites, 2006–2014. MMWR Morb Mortal Wkly Rep.

[9] Collado L, Gutiérrez M, González M, Fernández H (2013). Assessment of the prevalence and diversity of emergent campylobacteria in human stool samples using a combination of traditional and molecular methods. Diagn Microbiol Infect Dis.

[10] Fernández H, Kahler K, Salazar R, Ríos M (1994). Prevalence of thermotolerant species of *Campylobacter* and their biotypes in children and domestic birds and dogs in southern Chile. Rev Inst Med Trop Sao Paulo.

[11] Figueroa G, Galeno H, Troncoso M, Toledo S, Soto V (1989). Prospective study of *Campylobacter jejuni* infection in Chilean infants evaluated by culture and serology. J Clin Microbiol.

[12] Platts-Mills J, Kosek M (2014). Update on the burden of *Campylobacter* in developing countries. Curr Opin Infect Dis.

[13] Comité de Microbiología Clínica (Sociedad Chilena de Infectología), Laboratorio de Referencia de Bacteriología (Instituto de Salud Pública), Instituto de Ciencias Biomédicas (Facultad de Medicina, Universidad de Chile) (2002). Acute diarrheal syndrome: recommendations for the microbiological diagnosis. Rev Chilena Infectol.

[14] Chanqueo L, García P, León E, Blu F (2005). Evaluation of Hucker stain as *Campylobacter* sp screening detection during an acute diarrhea disease. Rev Chilena Infectol.

[15] Humphries R, Linscott A (2015). Laboratory diagnosis of bacterial gastroenteritis. Clin Microbiol Rev.

[16] On S (2013). Isolation, identification and subtyping of *Campylobacter*: where to from here?. J Microbiol Methods.

[17] Valenzuela ME, D’Ottone K (1984). Diagnóstico presuntivo rápido de enteritis asociada a *Campylobacter*. Rev Chil Infect.

[18] NCCLS (2004). Quality control for commercially prepared microbiological culture media; approved standard - third edition.

[19] Clinical and Laboratory Standards Institute (CLSI) (2015). Methods for antimicrobial dilution and disk susceptibility testing of infrequently isolated or fastidious bacteria.

[20] Sazie ES, Titus AE (1982). Rapid diagnosis of campylobacter enteritis. Ann Intern Med.

[21] Ho DD, Ault MJ, Ault MA, Murata GH (1982). *Campylobacter* enteritis: early diagnosis with Gram’s stain. Arch Intern Med.

[22] Park CH, Hixon DL, Polhemus AS, Ferguson CB, Hall SL, Risheim CC (1983). A rapid diagnosis of *Campylobacter* enteritis by direct smear examination. Am J Clin Pathol.

[23] Wang H, Murdoch DR (2004). Detection of *Campylobacter* species in faecal samples by direct Gram stain microscopy. Pathology.

[24] Mushi MF, Paterno L, Tappe D, Deogratius AP, Seni J, Moremi N (2014). Evaluation of detection methods for *Campylobacter* infections among under-fives in Mwanza City, Tanzania. Pan Afr Med J.

[25] Public Health England (2015). Identification of *Campylobacter* species. UK Standards for Microbiology Investigations. ID23 Issue 3. https://www.gov.uk/uk-standards-for-microbiology-investigations-smi-quality-and-consistency-in-clinical-laboratories. Accessed Dec. 29, 2015.

[26] Kist M, Neumeister B, Geiss HK, Braun RW, Kimmig P (2009). *Campylobacter* und *Arcobacter* spp. Mikrobiologische Diagnostik.

[27] Hurd S, Patrick M, Hatch J, Clogher P, Wymore K, Cronquist AB (2012). Clinical laboratory practices for the isolation and identification of *Campylobacter* in Foodborne Diseases Active Surveillance Network (FoodNet) sites: baseline information for understanding changes in surveillance data. Clin Infect Dis.

[28] Platts-Mills JA, Liu J, Gratz J, Mduma E, Amour C, Swai N (2014). Detection of *Campylobacter* in stool and determination of significance by culture, enzyme immunoassay, and PCR in developing countries. J Clin Microbiol.

[29] García P, Valenzuela N, Rodríguez M, León E, Fernández H (2009). Susceptibilidad antimicrobiana de *Campylobacter jejuni* aislado de coprocultivos en Santiago de Chile. Rev Chilena Infectol.

[30] Bemis K, Marcus R, Hadler J (2014). Socioeconomic status and campylobacteriosis, Connecticut, USA, 1999–2009. Emerg Infect Dis.

